# The Use of the Microscope in Clinical and Pathological Examinations

**Published:** 1885-11

**Authors:** 


**The Use of the Microscope in Clinical and Pathological Examinations.** By Dr. CARL FRIEDLAENDER, Privat-Docent in Pathological Anatomy at Berlin. Second Edition, enlarged and improved with a chromo lithograph. Translated by HENRY C. COE, Pathologist to Woman's Hospital. New York: D. Appleton & Co., No. 1 Bond street. Buffalo: Herger & Ulbrich. 1885.

Dr. Coe is to be commended for the excellent translation he has given us of this valuable work. A concise, but sufficiently complete, statement of the processes employed in pathological histology has been needed, for every one who aspires to follow the progress of pathology, must become acquainted with the recent methods of research. The technique of preparing cutting and staining specimens is given in a way that makes it possible for even a beginner to follow, and the expert will find in this book many a useful hint. The lithograph accompanying the book gives the various pathogenic schizomycetes, or, at least, the most important or characteristic of these which have been definitely isolated. They are specially useful for comparison, as they are all drawn on the same scale—one to one thousand.

